# Angiotensin II Type-1 Receptor Antibody in Solid Organ Transplantation – Is It Time to Test?

**DOI:** 10.3389/ti.2024.13280

**Published:** 2024-11-13

**Authors:** Paul James Patrick Martin, Michelle Willicombe, Candice Roufosse

**Affiliations:** ^1^ Department of Immunology and Inflammation, Imperial College London, Hammersmith Campus, London, United Kingdom; ^2^ Imperial College Renal and Transplant Centre, Imperial College Healthcare NHS Trust, Hammersmith Hospital, London, United Kingdom; ^3^ Department of Histopathology, Northwest London Pathology NHS Trust, Charing Cross Hospital, London, United Kingdom

**Keywords:** AT1R antibody, AT1R, non-HLA antibody, rejection, antibody mediated rejection

## Abstract

Angiotensin II type-1 receptor antibody (AT1R-Ab) has been mooted as a potential effector of both acute and chronic antibody mediated rejection (AMR). A growing body of literature on the topic is now coming under scrutiny in the context of the evolving Banff AMR diagnostic classification system and refinement of recommendations for histocompatibility testing by the Sensitization in Transplantation Assessment of Risk (STAR) workgroup. This mini-review discusses the latest understanding of pathophysiological mechanisms, clinical evidence for the pathogenicity of AT1R-Ab, and methods of laboratory testing.

## Introduction

Criteria for the diagnosis of antibody mediated rejection (AMR) in kidney allografts were first incorporated into the Banff Classification in 2001 and comprise both active and chronic components [1]. Classification criteria for AMR in other solid organ transplants have evolved at different rates [2–5]. Conventionally, donor human leucocyte antigen (HLA) proteins have been understood as the primary target of recipient alloimmune response, and are a major driver of late allograft rejection and loss [6]. However, as has become apparent in recent years, accounting for the HLA system alone is not the panacea for all immune-mediated transplant injury [7]. The latest Banff iteration has considered this gap in immunological understanding with the creation of a subcategory of C4d-negative microvascular inflammation/injury with absence of detectable circulating HLA donor-specific antibodies (HLA-DSA) [8].

In 2005, two significant papers on the concept of immune response in kidney transplantation were published. Opelz et al., in an international study of over 4,000 kidney transplant recipients from HLA-identical sibling donors, demonstrated that the presence of panel reactive antibodies >50% was associated with long-term allograft loss, suggesting that non-HLA antibodies may play a role in chronic rejection [9]. Separately, Dragun et al. reported the presence of agonistic IgG1 and IgG3 antibodies to angiotensin II type-1 receptor (AT1R) in the sera of kidney transplant recipients who had vascular rejection refractory to steroid treatment [10].

Since then, specific and sensitive tests for HLA-DSA have been developed and AT1R antibodies (AT1R-Ab) have become the most widely studied non-HLA antibody in transplantation, with conflicting reports on their association with allograft outcomes [11–14]. A comprehensive review of Dragun et al.’s contribution to our understanding of AT1R in transplantation was recently published [15]. This mini-review aims to highlight the latest research on pathophysiological mechanisms; to discuss methods of laboratory testing; and to outline current gaps in knowledge and potential for future research (Table 1).

**TABLE 1:** Future directions for research.

1. Development of a standardized, validated, high throughput, affordable method of testing
2. Collaboration across transplant disciplines to confirm or refute the association of AT1R-Ab with acute and/or chronic AMR and/or microvascular inflammation/injury, in retrospective and prospective observational cohorts, with an eye to determining a clinically meaningful threshold of AT1R-Ab positivity
3. Further investigate causality by reproducing and validating the mechanistic studies reported to date, and including specific investigation of the role that complement does or does not play in AT1R-Ab mediated pathology
4. Randomised controlled trials of therapeutic interventions that have thus far only been reported in case series

## Current Understanding of Pathophysiology of AT1R-Ab Mediated Rejection and Endothelial Injury

Angiotensin II, a potent vasoconstrictor that influences endothelial function, inflammation and fibrosis, primarily mediates its effect through AT1R, a G-protein coupled receptor (GPCR) [16–18]. Expression of AT1R is widespread but not ubiquitous, and concentrations on cell membranes fluctuate dependent on genetic and environmental factors [19]. AT1R-Ab function as receptor agonists [19]. They may be present at the time of organ transplantation, or develop *de novo* after transplantation.

Kidney transplant histological features in the context of AT1R-Ab positivity have been reported. Min et al. report that glomerulitis and peritubular capillaritis were the commonest biopsy findings amongst AT1R-Ab positive recipients [20]. In another cohort of 65 paediatric recipients, AT1R-Ab were associated with the presence of glomerulitis or arteritis [21]. In a prospective study, Lefaucheur et al. contemporaneously assessed AT1R-Ab and HLA-DSA serostatus at the time of indication and surveillance biopsies at or within 1 year of transplantation in 1,845 people. Recipients positive for HLA-DSA plus AT1R-Ab had the lowest allograft survival. Higher levels of circulating AT1R-Ab were associated with glomerultis, peritubular capillaritis, and intimal arteritis. Among recipients with histological rejection, AT1R-Ab positivity was associated with lower prevalence of complement deposition in peritubular capillaries (*p* < 0.001) [22]. This circumstantial evidence that AT1R-Ab can mediate vascular injury in a manner independent of complement corroborates the index cohort of Dragun et al., but is not a histological finding borne out uniformly in all studies.

Mechanistic studies have highlighted cellular signalling mechanisms influenced by AT1R-Ab. Catar et al. treated human microvascular cells with AT1R-Ab that had been isolated from seropositive patients with transplant vasculopathy. AT1R receptor signalling was sustained via beta2-arrestin recruitment to the cell membrane and mTOR complexes were activated with consequent impairment of endothelial repair capability [23]. These effects were terminated with pharmacological mTOR inhibition. Moll et al. determined that IgG derived from sera of kidney transplant recipients with vasculopathy stimulated secretion of tumour necrosis factor alpha from human microvascular endothelial cells with subsequent THP-1 monocyte activation [24]. The same effect was not demonstrated using IgG derived from the sera of a control cohort. Although the investigators do not explicitly state that AT1R-ab are implicated, this pro-inflammatory mechanism is proposed to act via GPCR-directed PAR1 signalling [24]. These *in vitro* models offer potential targets for therapeutic intervention.

## Clinical Studies of AT1R Antibodies in Solid Organ Transplantation

Most studies of AT1R-Ab have been undertaken in kidney transplant recipients (Table 2). In 2022, a meta-analysis by Kang et al of 21 studies concluded that recipients with AT1R-Ab were at greater risk of AMR (RR 1.96, 95% CI 1.61–2.33) and allograft failure (RR 2.37, 95% CI 1.50–3.75) [25]. The studies varied in size, but three, one of which has already been discussed [22], were notably larger. In a longitudinal study of 351 recipients, using positivity threshold >15 u/mL, Taniguchi reported that *de novo* AT1R-Ab and dual AT1R-Ab plus HLA-DSA positivity were associated with allograft loss [12]. Giral et al., in a cohort of 599 at a threshold of >10 U/mL, reported AT1R-Ab positivity in 47.2% of participants at time of transplantation, who had a 2.6 fold greater risk of allograft failure beyond 3 years [11]. Not included in the meta-analysis, Deltombe et al., using a positivity threshold of >10 u/mL, found no association between AT1R-Ab status and transplant outcomes in a cohort of 387 patients [13]. In 62 paediatric recipients, AT1R-Ab was associated with AMR using a positivity threshold of 9.5 u/mL [26]. More recently, two observational studies using positivity threshold of >17 u/mL did not show clear association with AMR [27, 28].

**TABLE 2:** Studies of angiotensin II type 1 receptor antibody (AT1R Ab) in solid organ transplantation.

Year	Authors	Study population	Transplantation period	AT1R Ab measurement technique	Measurement timepoints	ELISA AT1R Ab positivity threshold for analysis	Concurrent assessment of HLA DSA status	Main findings
2010	Reinsmoen et al.	97 adult kidney recipients	2006–2009	ELISA	Pre transplant Post transplant at time of rejection	>17 U/mL	Yes	1. AT1R Ab associated with AMR in the absence of HLA DSA
2012	Hiemann et al.	30 adult heart recipients	2005–2006	ELISA	Post transplant at multiple timepoints between 24 h and 1 year	>15.9 U/mL	Yes	1. AT1R Ab levels were higher in patients with rejection 2. AT1R Ab were associated with microvasculopathy
2013	Giral et al.	599 adult kidney recipients	1998–2007	ELISA	Pre transplant Post transplant	>10 U/mL	Yes	1. Higher pre transplant AT1R Ab correlated with allograft loss after 3 years
2013	Taniguchi et al.	351 adult kidney recipients	1999–2009	ELISA	Pre transplant Post transplant at multiple time points	>15 U/mL	Yes	1. Pathological synergy between AT1R Ab and HLA DSA 2. *de novo* AT1R Ab were associated with allograft failure
2014	Reinsmoen et al.	200 adult heart recipients	2007–2011	ELISA	Pre transplant Post transplant at multiple time points up to 1 year	>12 U/mL and >17 U/mL	Yes	1. Dual HLA DSA and AT1R Ab positivity was associated with lower freedom from rejection
2014	Ohe et al.	81 paediatric liver recipients	1990–2010	ELISA	Post transplant at time of indication or protocol biopsy	>17 U/mL	Yes	1. AT1R Ab were associated with allograft fibrosis in recipients who underwent withdrawal of immunosuppression
2016	Urban et al.	69 adult heart recipients *LVAD prior to transplant	2008–2014	ELISA	Prior to LVAD implantation Pre transplant	>17 U/mL	No	1. There was no difference in survival or freedom from rejection according to AT1R Ab status
2017	Deltombe et al.	940 adult kidney recipients	2008–2012	ELISA	Pre transplant only	>10 U/mL and >17 U/mL	Yes	1. AT1R Ab were not associated with higher risk of acute rejection episodes or allograft failure
2017	Reinsmoen et al.	162 adult lung recipients	2011–2013	ELISA	Pre transplant Post transplant at 3- and 6-months, and at time of allograft dysfunction	>11 U/mL and >17 U/mL	Yes	1. AT1R Ab were associated with lower freedom from rejection 2. AT1R Ab were associated with lower freedom from *de novo* HLA DSA
2017	Cozzi et al.	1 adult lung recipient	2015	ELISA	Pre transplant Day 4 post transplant	>10 U/mL	Yes	1. Case report highlighting possible implications of AT1R Ab
2017	O’Leary et al.	1,269 adult liver recipients	2000–2009	ELISA	Pre transplant 1 year post transplant	>17 U/mL	Yes	1. AT1R Ab and preformed HLA DSA were associated with increased mortality 2. *de novo* AT1R Ab were associated with rejection and fibrosis
2017	Gerlach et al.	29 adult intestine or multi-visceral recipients	2000–2015	ELISA	Pre transplant at time of listing and 3 monthly thereafter Post transplant weekly until hospital discharge and at time of allograft dysfunction	>12 U/mL	Yes	1. AT1R Ab were associated with rejection
2018	Min et al.	359 adult kidney recipients	2010–2014	ELISA	Pre transplant only	>10 U/mL	Yes	1. HLA DSA and AT1R Ab were associated with microvascular inflammation on biopsy 2. Positive AT1R-Ab status was associated with lower allograft survival
2018	Pearl et al.	65 paediatric kidney recipients	2005–2014	ELISA	Pre transplant Post transplant at multiple time points up to 2 years	>17 U/mL	Yes	AT1R Ab positivity is associated with: 1. Allograft loss 2. Lower eGFR over 2 years 3. Higher levels of TNF-alpha, IL-1beta, IL-8
2018	Fichtner et al.	62 paediatric kidney recipients	1999–2010	ELISA	Post transplant at time of indication biopsy	>9.5 U/mL	Yes	1. AT1R Ab positivity in context of indication biopsy was associated with AMR and reduced allograft function
2018	Kamburova et al.	87 healthy controls, 40 patients with miscellaneous kidney disease	Not applicable or not reported	Bead-based immunoassay	not reported	Not applicable	No	1. In house production of AT1R was not achieved 2. Testing for non-HLA Abs using multiplex platform is achievable
2019	Kamburova et al.	4,770 adult kidney recipients	1995–2006	Bead-based immunoassay	Pre transplant only	Not applicable	No	1. Main findings pertain to other non-HLA antibodies
2019	Lefaucheur et al.	1845 adult kidney recipients	2008–2012	ELISA	Post transplant within 1 year	>10 U/mL	Yes	AT1R Ab were associated with: 1. AMR at 1 year 2. diminished allograft survival 3. Microvascular inflammation on biopsy
2019	Delville et al.	48 adult kidney recipients	Not reported	ELISA and endothelial cell flow cytometry	Pre transplant only	>10 U/mL and >17 U/mL	Yes	1. AT1R Ab status failed to differentiate patients with microvascular inflammation in the absence of HLA DSA from stable patients 2. Cell based assays could improve risk assessment pre transplantation
2020	Villa et al.	1 adult heart recipient	Not reported	ELISA and endothelial cell flow cytometry	56 days pre transplant and day of transplant 27 days post transplant	Not applicable	Yes	1. Hyperacute fulminant allograft dysfunction in a recipient with markedly elevated AT1R Ab
2020	See et al.	64 adult heart recipients	1994–2014	ELISA	Post transplant	Not reported	Yes	1. There was no difference in AT1R Ab levels between two groups with and without AMR
2020	Wozniak et al.	79 paediatric liver recipients	2010–2017	ELISA	Post transplant at routine clinical visits or during allograft dysfunction	>17 U/mL	Yes	1. AT1R Ab prevalence was high in cases of allograft dysfunction
2020	Xu et al.	94 adult liver recipients	1991–2018	ELISA	Prior to second transplant	>17 U/mL and >40 U/mL	Yes	1. AT1R Ab were common in recipients of a second transplant 2. AT1R Ab were associated with inferior long-term outcomes
2021	Chan et al.	25 paediatric intestine recipients	2000–2016	ELISA	Post transplant at routine clinical visits or during allograft dysfunction	>17 U/mL	Yes	1. AT1R Ab were common post transplant 2. AT1R Ab were not associated with allograft dysfunction or survival
2021	Lamarthee et al.	389 adult kidney recipients	2012–2017	ELISA and endothelial cell flow cytometry	Pre transplant only	>10 U/mL	Yes	1. There was no correlation between immunoassay results and AT1R Ab levels 2. Pre transplant AT1R Ab did not correlate with increased risk for AMR
2022	Kang et al. *Systematic review of 21 studies	4,023 adult and paediatric kidney recipients	1998–2019	ELISA	Various	Various	Various	AT1R Ab positivity is associated with: 1. AMR (RR 1.96 CI 1.61–2.33) 2. Allograft loss (RR 2.37, CI 1.50–3.75)
2022	Pizzo et al.	36 paediatric kidney recipients	2011–2019	ELISA	Pre transplant Post transplant at multiple timepoints	>17 U/mL	Yes	1. AT1R Ab positivity was not associated with rejection or reduced allograft function
2022	Liu et al.	79 adult kidney recipients	2016–2019	ELISA	Pre transplant Post transplant within 1 year	>17 U/mL	Yes	1. AT1R Ab positivity was associated with AMR and lower EGFR
2022	Moreno et al.	21 adult heart recipients	2017–2019	ELISA	Post transplant at time of allograft dysfunction	>10 U/mL		1. AT1R Ab were associated with allograft dysfunction in the absence of rejection
2022	Senev et al.	874 adult kidney recipients	2004–2013	Bead-based immunoassay	Pre transplant Post transplant at 3 months, 1 year and 5 years	Not applicable	Yes	1. There was no association between pre-transplant AT1R Ab and occurrence of AMR
2023	Chou-Wu et al.	1 paediatric heart recipient	2007	ELISA	At time of first and second transplant, and post second transplant	Not applicable	Yes	1. Case report highlighting possible implications of AT1R Ab
2023	Son et al.	71 adult lung recipients	2016–2020	ELISA	Pre transplant Post transplant within 3 months	>17 U/mL	Yes	High AT1R Ab levels were associated with: 1. de novo HLA DSA 2. Acute cellular rejection 3. Early chronic lung allograft dysfunction 4. Lower recipient survival time
2024	Jung et al.	1 adult heart recipient	Not reported	ELISA	Pre transplant Post transplant at multiple time points	>17 U/mL	No	1. Case report highlighting possible implications of AT1R Ab

AMR, antibody mediated rejection; AT1R, Ab Angiotensin II type 1 receptor antibody; CI, 95% confidence interval; eGFR, estimated glomerular filtration rate; ELISA, enzyme linked immunosorbent assay; HLA DSA, human leucocyte antigen donor specific antibody; LVAD, left ventricular assist device; RR, relative risk.

In heart transplantation, a prospective study of 30 recipients demonstrated that persistently elevated AT1R-Ab levels were associated with AMR and microvasculopathy [29]. Elevated AT1R-Ab were reported in a cohort of 21 recipients with allograft dysfunction in the absence of AMR [30]. In a cohort of 200 recipients, concomitant *de novo* HLA-DSA and AT1R-Ab were associated with diminished freedom from AMR, and recipients with rising AT1R-Ab titres were more likely to be diagnosed with cardiac allograft vasculopathy (CAV) [31]. Characteristically, CAV does not often respond to conventional treatment of coronary artery disease and is a common cause of cardiac allograft failure [32]. In two studies of patients who underwent bridging to transplantation with a left ventricular assist device (LVAD), one reported that 63.8% of participants tested negative for AT1R-Ab initially and subsequently became positive post-LVAD, and another reported that higher titres of AT1R-Ab at time of transplant had worse outcomes [33, 34]. Vascular injury related to LVAD complements the auto-antigen exposure and auto-antibody recruitment theory previously described [35]. A number of illustrative case reports have been published in the literature: a fatal case of hyperacute AMR in a recipient with no HLA-DSA but high AT1R-Ab levels on the day of transplantation [36]; a paediatric case whereby a second allograft was lost in context of high AT1R-Ab [37]; and a case of AMR, CAV and persistently elevated AT1R-Ab levels despite repeated plasmapheresis, intravenous immunoglobulin and steroids [38]. Conversely, when testing for an array of 44 non-HLA antibodies in a cohort of 64 heart transplant recipients, 67% of whom had AMR, no association with AT1R-Ab was found [39]. In light of these findings, a recent Banff report specifically discussed the role of non-HLA antibodies such as AT1R-Ab in heart transplantation and advocated for development of standardized diagnostic tests, and prospective clinical trials to explore role in rejection and assess efficacy of treatments [3].

Regarding lung transplantation, in a multi-centre study of 162 recipients, 46% were positive for AT1R-Ab prior to transplantation, and frequency of *de novo* HLA-DSA and AMR was greater in those positive for AT1R-Ab [40]. In a cystic fibrosis patient, rapid onset AMR ensued in the absence of HLA-DSA but positive AT1R-Ab serostatus [41]. In 71 recipients, chronic lung allograft dysfunction at 3 years was more common in the AT1R-Ab positive group compared to the negative (58.3% vs. 11.8%, *p* < 0.001) [42].

Conventionally, liver allografts are perceived as lower immunological risk than other solid organ transplants. In 81 paediatric patients who received living donor transplants and subsequently had withdrawal of immunosuppression, AT1R-Ab >17 u/mL was evident in 65% of patients with advanced fibrosis, a greater proportion than those without fibrosis (*p* = 0.02) [43]. Two pertinent findings from a large study of 1,269 liver transplant recipients who had sera tested for AT1R-Ab and HLA-DSA, found that *de novo* AT1R-Ab was associated with increased risk of rejection and fibrosis progression, and histology from the *de novo* AT1R-Ab subgroup showed distinctive sinusoidal C4d staining spatially related to activated stellate cells [44]. In 79 paediatric recipients, those with active allograft dysfunction were more likely to be AT1R-Ab positive compared to those with stable function (89% vs. 29%, *p* = 0.001). Those with both AT1R-Ab and HLA-DSA were more likely to progress to allograft loss [45]. Regarding 94 patients receiving a second liver transplant, 51.1% had AT1R-Ab >17 u/mL at time of second transplant, and those with an AT1R-Ab level >40 u/mL were more likely to experience allograft loss [46].

As regards intestinal transplantation, in 29 recipients AMR was more common in those with positive AT1R-Ab versus those without (55% vs. 11%, *p* < 0.01) [47]. In 25 paediatric recipients, 68% had AT1R-Ab >17 u/mL pre-transplant; these levels did not vary significantly when sera were tested sequentially, and there was no association with allograft dysfunction. No explicit comment was made regarding AMR [48].

## Current Techniques in Testing for AT1R-Ab and Related Problems

The most commonly used testing platform for AT1R-Ab is the enzyme linked immunosorbent assay (ELISA) available from CellTrend GmbH Luckenwalde Germany which can test 40 serum samples in 1 run. AT1R is pre-coated on the microtiter plate. During the first incubation, AT1R-Ab in samples are immobilized on the plate and detected with labelled anti-human IgG. The intensity of the colour in the subsequent enzymatic substrate reaction correlates with the concentration of AT1R-Ab. Reinsmoen et al. were the first to use this technique in a clinical study [49].

Senev et al. used the solid-phase Luminex assay to retrospectively test pre- and post-transplantation sera of 874 recipients for 82 different non-HLA antibodies including AT1R-Ab. There was an association between the burden of pretransplant non-HLA antibodies and development of AMR without HLA-DSA (HR 1.3 per 10 antibodies *p* = 0.02) and microvascular inflammation (HR 1.13 per 10 antibodies *p* = 0.04). Only four antigens were identified as independent risk factors for AMR histology, AT1R was not among them [50]. Kamburova et al created a solid-phase assay to test sera for 14 specific non-HLA antibodies. Production of AT1R proteins was hampered by protein cleavage before excretion in the culture supernatant, therefore AT1R-Ab was largely excluded from reported results [51]. Nevertheless, in a study by the same group, AT1R was included as an antigen in the same assay screening for non-HLA antibodies in pre-transplant sera of 4,770 recipients; no association between AT1R-Ab status and graft survival was observed [14].

Alternatives to solid-phase assays have been reported. Delville et al. used a cell-based crossmatch assay to identify pre-formed IgG antibodies to glomerular endothelial cells in a small cohort of highly selected transplant recipients. All participants had microvascular inflammation on early biopsies but no circulating HLA-DSA; 26% were positive for AT1R-Ab at threshold 10 U/mL [52]. Lamarthee et al. refined this strategy by using CRISPR/Cas9 to render glomerular endothelial cells devoid of HLA -A-B-C & -DR expression to develop a non-HLA antibody detection immunoassay (NHADIA). In an unselected cohort of 389 recipients, pre-transplant NHADIA values were associated with AMR histology (*p* = 0.0082) and microvascular inflammation (0.0024). However, using a positivity threshold of 10 U/mL and presumably measured using ELISA technique, there was no correlation between AT1R-Ab levels and NHADIA values [53]. In a novel approach, Lammerts et al. isolated endothelial cells from the perfusion fluid of 102 donor kidneys and propagated a biobank of machine perfusion-derived primary renal endothelial cells (MP-PRECs) to primarily study anti-HLA mediated cytotoxicity, but noted the technique could have utility for investigating non-HLA antibody mediated disease also [54]. Of course, in the context of studying AT1R-Ab, this will be contingent on MP-PRECs expressing AT1R.

## Current Research Gaps and Potential Future Directions

The role of AT1R-Ab in transplantation continues to be investigated. *In vitro* mechanistic studies have outlined how AT1R-Ab influence signalling pathways to cause vascular injury. A steadily growing number of small to medium sized clinical studies highlight a link between AT1R-Ab status, both pre-transplant and *de novo*, and development of AMR, with possible pathological synergy in cases of concomitant HLA-DSA positivity. These studies are hampered by heterogeneous study design and inconsistent outcome reporting with variable thresholds of antibody positivity. A large, prospective two-centre study using solid-phase ELISA assay firmly established a link between AT1-Ab and a phenotype of AMR, but this link has not been borne out in similarly large retrospective studies using cell-based assays. There is no explanation for this discordance at present. The Bradford Hill criteria should be borne in mind when considering these and future studies investigating the role of non-HLA antibodies in causality of AMR [55].

Regarding ELISA specifically, a particular advantage is the capacity to bulk test; for instance, screening the stored sera of a transplant wait-list population. There are a number of disadvantages. For interpolation of results, a standard curve must be created for each ELISA kit. This introduces a degree of inter-assay variability making comparison of results between kits difficult. If antibody titres in a sample are high and the result is beyond the upper limit of the standard curve, the test needs to be performed again at greater dilution factor to obtain a discrete concentration value. Furthermore, as highlighted by Kamburova et al., details of the manufacturing processes of commercially available ELISA assays are obscure, preventing in-house replication of these assays and necessary reagents [51].

In heart transplantation, CAV is considered a manifestation of chronic rejection and is associated with both non-HLA antibodies and HLA-DSA [56]. As a medium-sized vasculopathy, it is possible that CAV is analogous with some types of the morphologically heterogenous radiologic abnormality of transplant renal artery stenosis (TRAS). Kidney recipients with a post-anastomotic TRAS lesion are more likely to have *de novo* class II HLA-DSA, suggesting a possible immune-mediated pathological process for some [57]. No studies assessing the relationship between AT1R-Ab and TRAS have been performed. In liver transplantation, reports of a distinctive histological pattern of sinusoidal C4d staining are in contrast to the reports of C4d negativity in many kidney studies. Mechanistic studies to date have elucidated injurious aberrations to cell-signalling pathways, but perhaps it is too soon to discount a potential complement-mediated mechanism also.

The creation in the Banff 2022 report of the descriptive phenotype microvascular injury, C4d-negative, anti-HLA DSA-negative has focussed the minds of researchers on finding a cause. The burgeoning number of non-HLA antibodies that have been studied and subsequently implicated in allograft injury represents both an opportunity and a challenge for transplantation medicine. Given the broad array of potential antigenic targets, there has been no recommendation to test for specific non-HLA antibodies as part of a transplant recipient’s immunological evaluation [58]. The Sensitization in Transplantation: Assessment of Risk (STAR) workgroup, initially established to aggregate cross-discipline knowledge on HLA histocompatabilty, has turned its attention to non-HLA antibodies in its latest report [59]. Acknowledging that studies to date have not firmly established temporal causality between AT1R-Ab status and development of AMR, the report nevertheless advocates for the development of standardized high-throughput testing for non-HLA antibodies [59]. Indeed, given that treatment options such as pharmacological receptor blockade and plasmapheresis have been reported as beneficial, it could be argued that access to validated testing for AT1R-Ab is an unmet clinical need for transplant recipients. For strategies to mitigate the potential pathologic effects of non-HLA antibodies in transplantation, readers are referred to the review by Kardol-Hoefnagel and Otten [60].

Large prospective studies are likely to be required to determine the clinically significant threshold of AT1R-Ab positivity. To take a pragmatic approach, expanding testing capabilities for patients on the transplant wait list first could be prioritised to allow us to better understand the epidemiological burden of AT1R-Ab positivity prior to transplantation, stratify patients at risk of developing chronic rejection related to AT1R-Ab, and identify recruits to prospective randomised controlled trials of therapeutic intervention.
