# Supplementary material for: Angiotensin II Type-1 Receptor Antibody in Solid Organ Transplantation – Is It Time to Test?
Source: Transpl Int. 2024 Nov 13;37:13280. doi: 10.3389/ti.2024.13280 (PMC11598415; doi:10.3389/ti.2024.13280)
Supplement: Supplementary file 1 [file Table1.DOCX]

**Table one**

Future Directions for Research

1. Development of a standardized, validated, high throughput, affordable method of testing.
2. Collaboration across transplant disciplines to confirm or refute the association of AT1R-Ab with acute and/or chronic AMR and/or microvascular inflammation/injury, in retrospective and prospective observational cohorts, with an eye to determining a clinically meaningful threshold of AT1R-Ab positivity.
3. Further investigate causality by reproducing and validating the mechanistic studies reported to date, and including specific investigation of the role that complement does or does not play in AT1R-Ab mediated pathology.
4. Randomised controlled trials of therapeutic interventions that have thus far only been reported in case series.
