# Supplementary material for: Angiotensin II Type-1 Receptor Antibody in Solid Organ Transplantation – Is It Time to Test?
Source: Transpl Int. 2024 Nov 13;37:13280. doi: 10.3389/ti.2024.13280 (PMC11598415; doi:10.3389/ti.2024.13280)
Supplement: Supplementary file 2 [file Table2.DOCX]

| **Table Two. Studies of Angiotensin II Type 1 Receptor Antibody (AT1R Ab) in solid organ transplantation** | | | | | | | | |
| --- | --- | --- | --- | --- | --- | --- | --- | --- |
| **Year** | **Authors** | **Study population** | **Transplantation period** | **AT1R Ab measurement technique** | **Measurement timepoints** | **ELISA AT1R Ab positivity threshold for analysis** | **Concurrent assessment of HLA DSA status** | **Main findings** |
| 2010 | Reinsmoen et al | 97 adult kidney recipients | 2006 - 2009 | ELISA | Pre transplant Post transplant at time of rejection | >17 U/ml | yes | 1. AT1R Ab associated with AMR in the absence of HLA DSA |
| 2012 | Hiemann et al | 30 adult heart recipients | 2005 - 2006 | ELISA | Post transplant at multiple timepoints between 24hours and 1 year | >15.9 U/ml | yes | 1. AT1R Ab levels were higher in patients with rejection  2. AT1R Ab were associated with microvasculopathy |
| 2013 | Giral et al | 599 adult kidney recipients | 1998 - 2007 | ELISA | Pre transplant  Post transplant | >10 U/ml | yes | 1. Higher pre transplant AT1R Ab correlated with allograft loss after 3 years |
| 2013 | Taniguchi et al | 351 adult kidney recipients | 1999 - 2009 | ELISA | Pre transplant Post transplant at multiple time points | >15 U/ml | yes | 1. Pathological synergy between AT1R Ab and HLA DSA  2. de novo AT1R Ab were associated with allograft failure |
| 2014 | Reinsmoen et al | 200 adult heart recipients | 2007 - 2011 | ELISA | Pre transplant Post transplant at multiple time points up to 1 year | >12 U/ml and >17 U/ml | yes | 1. Dual HLA DSA and AT1R Ab positivity was associated with lower freedom from rejection |
| 2014 | Ohe et al | 81 paediatric liver recipients | 1990 - 2010 | ELISA | Post transplant at time of indication or protocol biopsy | >17 U/ml | yes | 1. AT1R Ab were associated with allograft fibrosis in recipients who underwent withdrawal of immunosuppression |
| 2016 | Urban et al | 69 adult heart recipients *LVAD prior to transplant | 2008 - 2014 | ELISA | Prior to LVAD implantation  Pre transplant | >17 U/ml | no | 1. There was no difference in survival or freedom from rejection according to AT1R Ab status |
| 2017 | Deltombe et al | 940 adult kidney recipients | 2008 - 2012 | ELISA | Pre transplant only | >10 U/ml and >17 U/ml | yes | 1. AT1R Ab were not associated with higher risk of acute rejection episodes or allograft failure |
| 2017 | Reinsmoen et al | 162 adult lung recipients | 2011 - 2013 | ELISA | Pre transplant  Post transplant at 3- and 6-months, and at time of allograft dysfunction | >11 U/ml and >17 U/ml | yes | 1. AT1R Ab were associated with lower freedom from rejection 2. AT1R Ab were associated with lower freedom from de novo HLA DSA |
| 2017 | Cozzi et al | 1 adult lung recipient | 2015 | ELISA | Pre transplant  Day 4 post transplant | > 10 U/ml | yes | 1. Case report highlighting possible implications of AT1R Ab |
| 2017 | O'Leary et al | 1269 adult liver recipients | 2000 - 2009 | ELISA | Pre transplant 1 year post transplant | >17 U/ml | yes | 1. AT1R Ab and preformed HLA DSA were associated with increased mortality 2. de novo AT1R Ab were associated with rejection and fibrosis |
| 2017 | Gerlach et al | 29 adult intestine or multi-visceral recipients | 2000 - 2015 | ELISA | Pre transplant at time of listing and 3 monthly thereafter  Post transplant weekly until hospital discharge and at time of allograft dysfunction | >12 U/ml | yes | 1. AT1R Ab were associated with rejection |
| 2018 | Min et al | 359 adult kidney recipients | 2010 - 2014 | ELISA | Pre transplant only | >10 U/ml | yes | 1. HLA DSA and AT1R Ab were associated with microvascular inflammation on biopsy 2. Positive AT1R-Ab status was associated with lower allograft survival |
| 2018 | Pearl et al | 65 paediatric kidney recipients | 2005 - 2014 | ELISA | Pre transplant Post transplant at multiple time points up to 2 years | >17 U/ml | yes | AT1R Ab positivity is associated with: 1. Allograft loss 2. Lower eGFR over 2 years 3. Higher levels of TNF-alpha, IL-1beta, IL-8 |
| 2018 | Fichtner et al | 62 paediatric kidney recipients | 1999 - 2010 | ELISA | Post transplant at time of indication biopsy | >9.5 U/ml | yes | 1. AT1R Ab positivity in context of indication biopsy was associated with AMR and reduced allograft function |
| 2018 | Kamburova et al | 87 healthy controls, 40 patients with miscellaneous kidney disease | Not applicable or not reported | Bead-based immunoassay | not reported | Not applicable | no | 1. In house production of AT1R was not achieved 2. Testing for non-HLA Abs using multiplex platform is achievable |
| 2019 | Kamburova et al | 4770 adult kidney recipients | 1995 - 2006 | Bead-based immunoassay | Pre transplant only | Not applicable | no | 1. Main findings pertain to other non-HLA antibodies |
| 2019 | Lefaucheur et al | 1845 adult kidney recipients | 2008 - 2012 | ELISA | Post transplant within 1 year | >10 U/ml | yes | AT1R Ab were associated with: 1. AMR at 1 year 2. diminished allograft survival 3. Microvascular inflammation on biopsy |
| 2019 | Delville et al | 48 adult kidney recipients | Not reported | ELISA and endothelial cell flow cytometry | Pre transplant only | >10 U/ml and >17 U/ml | yes | 1. AT1R Ab status failed to differentiate patients with microvascular inflammation in the absence of HLA DSA from stable patients 2. Cell based assays could improve risk assessment pre transplantation |
| 2020 | Villa et al | 1 adult heart recipient | Not reported | ELISA and endothelial cell flow cytometry | 56 days pre transplant and day of transplant 27 days post transplant | Not applicable | yes | 1. Hyperacute fulminant allograft dysfunction in a recipient with markedly elevated AT1R Ab |
| 2020 | See et al | 64 adult heart recipients | 1994 - 2014 | ELISA | Post transplant | Not reported | yes | 1. There was no difference in AT1R Ab levels between two groups with and without AMR |
| 2020 | Wozniak et al | 79 paediatric liver recipients | 2010 - 2017 | ELISA | Post transplant at routine clinical visits or during allograft dysfunction | >17 U/ml | yes | 1. AT1R Ab prevalence was high in cases of allograft dysfunction |
| 2020 | Xu et al | 94 adult liver recipients | 1991 - 2018 | ELISA | Prior to second transplant | >17 U/ml and >40 U /ml | yes | 1. AT1R Ab were common in recipients of a second transplant 2. AT1R Ab were associated with inferior long-term outcomes |
| 2021 | Chan et al | 25 paediatric intestine recipients | 2000 - 2016 | ELISA | Post transplant at routine clinical visits or during allograft dysfunction | >17 U/ml | yes | 1. AT1R Ab were common post transplant 2. AT1R Ab were not associated with allograft dysfunction or survival |
| 2021 | Lamarthee et al | 389 adult kidney recipients | 2012 - 2017 | ELISA and endothelial cell flow cytometry | Pre transplant only | >10 U/ml | yes | 1. There was no correlation between immunoassay results and AT1R Ab levels 2. Pre transplant AT1R Ab did not correlate with increased risk for AMR |
| 2022 | Kang et al  *Systematic review of 21 studies | 4023 adult and paediatric kidney recipients | 1998 -2019 | ELISA | Various | Various | various | AT1R Ab positivity is associated with: 1. AMR (RR 1.96 CI 1.61 - 2.33) 2. Allograft loss (RR 2.37, CI 1.50 - 3.75) |
| 2022 | Pizzo et al | 36 paediatric kidney recipients | 2011 - 2019 | ELISA | Pre transplant  Post transplant at multiple timepoints | >17 U/ml | yes | 1. AT1R Ab positivity was not associated with rejection or reduced allograft function |
| 2022 | Liu et al | 79 adult kidney recipients | 2016 - 2019 | ELISA | Pre transplant  Post transplant within 1 year | >17 U/ml | yes | 1. AT1R Ab positivity was associated with AMR and lower EGFR |
| 2022 | Moreno et al | 21 adult heart recipients | 2017 - 2019 | ELISA | Post transplant at time of allograft dysfunction | >10 U/ml |  | 1. AT1R Ab were associated with allograft dysfunction in the absence of rejection |
| 2022 | Senev et al | 874 adult kidney recipients | 2004 - 2013 | Bead-based immunoassay | Pre transplant Post transplant at 3 months, 1 year and 5 years | Not applicable | yes | 1. There was no association between pre-transplant AT1R Ab and occurrence of AMR |
| 2023 | Chou-Wu et al | 1 paediatric heart recipient | 2007 | ELISA | At time of first and second transplant, and post second transplant | Not applicable | yes | 1. Case report highlighting possible implications of AT1R Ab |
| 2023 | Son et al | 71 adult lung recipients | 2016 - 2020 | ELISA | Pre transplant  Post transplant within 3 months | >17 U/ml | yes | High AT1R Ab levels were associated with: 1. de novo HLA DSA 2. Acute cellular rejection 3. Early chronic lung allograft dysfunction 4. Lower recipient survival time |
| 2024 | Jung et al | 1 adult heart recipient | Not reported | ELISA | Pre transplant Post transplant at multiple time points | >17 U/ml | no | 1. Case report highlighting possible implications of AT1R Ab |

AMR Antibody mediated rejection

AT1R Ab Angiotensin II type 1 receptor antibody

CI 95% confidence interval

eGFR Estimated glomerular filtration rate

ELISA Enzyme linked immunosorbent assay

HLA DSA Human leucocyte antigen donor specific antibody

LVAD Left ventricular assist device

RR Relative risk
